# Beyond hypertension: integrated cardiovascular care as a path to comprehensive primary care

**DOI:** 10.2471/BLT.17.197996

**Published:** 2017-12-20

**Authors:** Sandeep P Kishore, David J Heller, Ashwin Vasan

**Affiliations:** a Arnhold Institute for Global Health, Icahn School of Medicine at Mount Sinai Health System, 1216 Fifth Avenue, Room 562, New York, New York 10029, United States of America (USA).; b Department of Population & Family Health, Columbia University Mailman School of Public Health, New York, USA.; Correspondence to Sandeep P Kishore (email: sunny.kishore@gmail.com).

At the United Nations General Assembly in 2016, the World Health Organization (WHO) and the Centers for Disease Control and Prevention in the United States of America launched Global Hearts, an initiative that includes the HEARTS technical package for cardiovascular disease management in primary health care.^1^ Building on WHO’s package of essential noncommunicable disease interventions, Global Hearts focuses on the noncommunicable disease burden due to atherosclerotic cardiovascular disease and its risk factors. The initiative also provides a simple framework that enables front-line health clinics to implement WHO’s longstanding call to integrate cardiovascular disease care into primary disease prevention.^2^ In addition, Global Hearts includes the building blocks for ending vertical, disease-specific approaches to cardiovascular conditions. Instead, it guides the organization and delivery of cardiovascular disease care for the whole patient through better screening, treatment and prevention. Given that cardiovascular disease is the principal cause of death worldwide and is increasingly common, tackling the disease is crucial for achieving meaningful primary care. However, to do so successfully, the initiative needs to extend cardiovascular disease care to include conditions such as heart failure and rheumatic heart disease. More importantly, Global Hearts must be incorporated into existing health-care delivery systems and into the public health laws and policies crucial for primary disease prevention.

Global Hearts’ approach to disease control includes the testing and treatment of several disease risk factors. Although the list of noncommunicable diseases causing most human illness and death is long, there are only a few risk factors for the four main conditions: heart disease, diabetes, cancer and lung disease. The initiative deals with them all, at least within the health sector. Its treatment protocols involve training health workers to assess and manage unhealthy diets, tobacco use and sedentary behaviour in healthy people, thereby helping prevent not only cardiovascular disease, but also cancer and metabolic disease. Yet, unlike initiatives claiming to fight all noncommunicable diseases or even all human diseases, Global Hearts has a specificity and focus that can galvanize donors and policy-makers and make noncommunicable diseases more visible. The global approach adopted to the human immunodeficiency virus (HIV) epidemic demonstrates how an aggressive, disease-specific campaign can reduce the global disease burden. Measurable indicators enable funders to evaluate the impact of initiatives on death and disease. In an era of public health austerity with a focus on the return on investment, the rationale for this approach is clear: distilling a broad campaign into several specific core goals gets both more support and faster results.

Child survival programmes provide a useful analogy. Over two decades ago, efforts to improve child survival coalesced around three programmes: the GOBI growth monitoring, oral rehydration, breastfeeding and immunization) programme and programmes on nutrition and malaria.^3^ Subsequently, these vertical initiatives were combined by WHO into the Integrated Management of Childhood Illness, which resulted in evidence-based, scalable, cost–effective protocols that were widely adopted.

Despite being the leading killers of adults worldwide, noncommunicable diseases have struggled to attract sufficient funding, that is, more than the current 1% of development assistance for health. Rather than forming overnight coalitions to address disparate conditions, noncommunicable disease practitioners could learn from the GOBI approach:^4^ define the core problems, then prove you can tackle them cheaply and feasibly. An integrated programme for the remaining problems will follow, with financial support to match. The Global Hearts initiative’s clear focus on cardiovascular disease is an excellent start.

Nevertheless, Global Hearts’ streamlined approach currently overlooks some major contributors to cardiovascular disease, including conditions readily treatable by non-physician, front-line health-care workers. For example, in sub-Saharan Africa, congestive heart failure is far more common than coronary artery disease because of the prevalence of rheumatic heart disease, hypertensive heart disease and other risk factors.^5^ The organization Partners in Health and others have demonstrated that nurses can be trained to screen for, and manage, heart failure, and even to carry out echocardiography.^6^ In the absence of this training, rheumatic heart disease can be prevented by benzathine penicillin injection or oral penicillin V.^7^ Providing basic guidelines on heart failure prevention and treatment would increase Global Hearts’ impact. Moreover, such guidelines may attract attention from partner countries and donors: at present, the large rheumatic disease burden confronted by many low- and middle-income countries means they would be unable to embrace any programme that fails to address this condition.^5^

More broadly, to work in practice, Global Hearts must be better integrated with local health systems and the underlying policy ecosystem. Primary care practitioners everywhere know that targeted interventions can pose challenges when implemented in resource-poor settings: at best they neglect patients’ other needs and at worst they neglect or undermine the existing health infrastructure. This is particularly true in the lowest-income countries where even nurse-led primary care facilities are often absent and care is provided only by trained laypersons, such as community health workers.

Take hypertension, for instance. We now know that about half of hypertension-related deaths could be averted through adherence to antihypertensive treatment.^7^ We also know that community health workers with minimal medical training can be as effective as physicians in helping patients lower their blood pressure through counselling and the supervised prescription of medicines.^8^ Moreover, the cost would be substantially less than for treating HIV and other infectious diseases. Therefore, investment in hypertension treatment can and does have a large, sustained impact at a low cost. However, hypertension is most harmful to people with other diseases and risk factors, such as diabetes, a high cholesterol level and tobacco use. To ignore these cofactors is to fail to treat the whole patient and to miss opportunities for controlling cardiovascular disease in the most vulnerable. Yet, programmes tackling all these factors can quickly grow too complex for health workers to implement alongside many other competing disease programmes. In implementing Global Hearts, therefore, clarity is needed on how fighting its target diseases, such as hypertension and obesity, can be achieved within a local health system focused instead on conditions like malaria and pneumonia.

It is a difficult balance: vertical programmes (e.g. smallpox eradication or HIV control) can inspire donors, but risk undermining other disease control efforts, whereas too broad an approach risks losing donors entirely. The Global Hearts initiative provides some tools to strike the balance of being both broad and specific. For example, it includes a 10-year risk stratification tool produced by WHO and the International Society for Hypertension that can be used to easily determine who would benefit most from cardiovascular disease treatment.^9^ The tool takes into account not only blood pressure but also tobacco use, diabetes status, cholesterol levels and other factors. People with a 10-year cardiovascular disease risk greater than 30% receive statins regardless of cholesterol level, thereby preventing stroke and peripheral arterial disease in addition to coronary atherosclerosis. As well, everyone is counselled on smoking cessation, thereby preventing atherosclerotic cardiovascular disease, Global Hearts’ focus, but incidentally targeting conditions such as cancer and lung disease. However, unlike WHO’s package of essential noncommunicable disease interventions, Global Hearts does not include policies known to deter smoking and reduce hypertension in the first place despite clear evidence of their impact and cost-effectiveness.

An unresolved question is of how cardiovascular disease care algorithms can be applied consistently by community health workers, who are already overloaded by competing tasks such as vaccination and contraception counselling. Modern evidence and best practices also evolve rapidly placing more burdens on health workers. For instance, especially when the evidence underlying some Global Hearts’ interventions (e.g. the selective use of statins for primary cardiovascular disease prevention) is evolving rapidly.^10^ Answering this question will require rigorous pilot projects and careful process evaluation. In particular, research is needed to prove that the Global Hearts’ protocols can be integrated with, and not undermine, existing primary care frameworks in low- and middle-income countries. To date, preliminary research has been carried out by Médecins Sans Frontières and Oxford University in Jordan^11^ and by the Integrated Management of Adolescent and Adult Illness Alliance in Uganda (Gove S and Shah M, Integrated Management of Adolescent and Adult Illness Alliance, personal communication, 2017). 

If the Global Hearts initiative achieves an integrated approach to cardiovascular disease care, primary care will be strengthened in several ways. First, Global Hearts can provide an opportunity for screening healthy people for an asymptomatic, but deadly disease. The initiative can help shift the health systems of low- and middle-income countries away from providing an acute, episodic service towards long-term, preventive care that can track patients over time for many cardiovascular conditions. Second, Global Hearts can encourage the use of integrated tools such as fixed-dose combination medications (i.e. polypills) that can treat several conditions simply and effectively while improving adherence and outcomes and avoiding supply shortages, a key obstacle to primary and secondary prevention. Third, Global Hearts can stimulate the introduction of information technology and tools to track progress, which will promote better access to therapy, improve outcomes and expand the population covered. Finally, this approach to care, especially if it involves policy interventions and cost–effectiveness research, will establish a low-cost, scalable model of care for noncommunicable diseases that is in line with sustainable development goal 3.

For HIV, the adoption of a similar, primary care-driven model of care, which emphasized decentralization, simplification and task-shifting, resulted in antiretroviral therapy coverage for over 90% of those in need in some situations.^12^ With the Global Hearts initiative, an analogous, primary care-based, integrated approach to cardiovascular disease can shift the previous focus on hypertension towards broader global goals.
